# Quartz Crystal Microbalance Technology Coupled with Impedance for the Dynamic Monitoring of the Cardiomyocyte Beating Function and Drug Screening

**DOI:** 10.3390/bios13020198

**Published:** 2023-01-28

**Authors:** Zhen Zhou, Xiaoyu Zhang, Tiean Zhou, Fushen Huang, Jinjun Chen

**Affiliations:** 1 College of Bioscience and Biotechnology, Hunan Agricultural University, Changsha 410128, China; 2 Hunan Provincial Engineering Technology Research Center for Cell Mechanics and Function Analysis, Changsha 410128, China; 3 College of Veterinary Medicine, Hunan Agricultural University, Changsha 410128, China

**Keywords:** electrical impedance sensing, quartz crystal microbalance, interface modification, condition optimization, association, rat primary coronary

## Abstract

The main sensing techniques used to study myocardial pulsation are electrical impedance sensing (EIS) and by quartz crystal microbalance (QCM). While electrical impedance technology is the gold standard for the study of myocardial pulsation, the clinical application of drugs is being followed up in real time additionally, thus, QCM technology needs to be further developed as a very important class of quality sensor technology. Moreover, the application of EIS, in combination with the QCM, for monitoring myocardial pulsation, has been rarely reported. In this paper, a series of cell growth and adhesion conditions were optimized using rat primary cardiomyocytes, and QCM was used in combination with EIS to monitor the adhesion and the myocardial pulsation ability of the cells in real time. Furthermore, cardiomyocytes that adhered to the QCM and EIS were treated with isoprenaline (ISO), a positive inotropic drug, and verapamil (VRP), a negative inotropic drug. Next, the cell index (CI)-time (T) plots, beating amplitude (BA) and beating rate (BR) of the cardiomyocytes were calculated and changes in these parameters, before and after, dosing were evaluated. The results showed that the QCM technique results were not only consistent with the results obtained with EIS, but also that the QCM technique had a certain degree of sensitivity for the calculation of cardiomyocyte beating. Thus, our findings validate the reliability and validity of the QCM technique for measuring cardiomyocyte beating and drug testing. We hope that further studies would evaluate the application of the QCM technology for clinical use.

## 1. Introduction

Drug-induced cardiotoxicity has emerged as one of the major causes of drug withdrawal and drug attrition in recent decades [1,2]. On the one hand, drug-induced cardiotoxicity threatens people’s health and even their lives. On the other hand, drug development is a very long process with a low success rate. It typically takes 12 years from preclinical testing to the final FDA approval of a drug, costing more than USD 1 billion in total [3]. Therefore, there is an urgent need to develop preclinical screening methods for the early prediction of drug-induced cardiotoxicity and reduce drug attrition, as well as health risks, in clinical applications [4,5]. Based on the specific properties of cardiomyocytes, many tools or methods have been developed to characterize the physiological parameters of cardiomyocytes. For example, Wang Tianxing et al. [6] designed and processed impedance sensors for the simultaneous detection of cardiomyocyte growth and beating, enabling the application of such cardiomyocyte sensors for the validation of drug effects on the ion channels and the related drug safety analysis. Wang Shuyan et al. [7] established a real-time cell analysis system (RTCA) to measure cardiotoxicity and applied it to screen for the early signs of drug induced cardiotoxicity. Qingjun Liu et al. [8] developed a novel cell sensor that could be used for detecting cardiomyocyte beating using an optical addressable potential sensor, and investigated the potential application of the sensor for screening and analyzing the active ingredients of drugs, and used typical cardiovascular inhibitory drugs (carbachol) and agonists (isoproterenol) as the proof-of-concept. Nevertheless, these methods generally assess the drug efficacy and toxicity by detecting changes in the various physiological parameters of cells, such as the cellular impedance, extracellular potential, extracellular acidification rate or changes in the generated extracellular potential and cellular morphology, as well as by observing changes in the cardiomyocyte beat frequency, amplitude and beat rhythm irregularity (BRI) index. The QCM is an advanced technology, based on the resonant electromechanical coupling model of piezoelectric quartz crystals, which uses the principle of the piezoelectric effect to convert small mass changes on the electrodes into frequency changes through certain conversion methods, and displays them on a monitor. It is a very versatile sensor device [9,10], which simultaneously measures the changes in multiple physicochemical properties of the cellular system [11,12]. In recent years, QCMs have also been applied to study cardiomyocytes. For instance, Li et al. [13,14] coupled the QCM with a microfluidic chip and enabled the tracking of the frequency changes in the systolic and diastolic processes of individual cardiomyocytes, by adding an adult rat cardiomyocyte to a 5 MHz quartz crystal through a microfluidic chip. Angelika Kunze et al. [15] monitored the effect of conventional drugs (E-4031 or nifedipine) on the QT interval of cardiomyocytes and their mechanical properties using QCM-D. The above studies showed that the QCM could detect the contractile mechanical force of cardiomyocytes, regardless of the alterations in the potential of the extracellular field, which has the great potential for drug screening. The group pioneered a QCM-based method for the quantitative determination of cellular forces, which was successively published as a Chinese invention patent and a PCT international patent. In this study, we focused on the application of the QCM technology to monitor the beating function of rat primary cardiomyocytes, without a specific force analysis. Since electrical impedance technology has achieved a lot of results as the gold standard in the study of myocardial pulsatile function [16,17], and allows the evaluation of the clinical application of drugs in real time, we aimed to combine the EIS and QCM techniques. With this, we sought to compare the myocardial pulsation signals obtained from the real-time monitoring of image scans on the QCM, with pulsation signals obtained from EIS, to establish the basis for the QCM monitoring of the dynamic changes in the pulsation and mechanical parameters of primary cardiomyocytes. In order to obtain the optimal myocardial beat signal, a series of prior selections were made, with respect to the cell density of cardiomyocytes loaded on the EIS, the self-assembly modification of the chip and the thickness of the chip. However, the pattern of changes in the beat frequency and beat amplitude, occurring before and after dosing of cells on a quartz crystal microbalance, need to be investigated by future studies, which may enable the clinical application of the above combination.

## 2. Apparatus and Reagents

### 2.1. Animals

Newborn 2-day-old SD rats were purchased from Hunan Slaughter Jingda Experimental Animals Co., Changsha, Hunan and used in our study.

### 2.2. Main Reagents and Drugs

DMEM (Dulbecco’s Modified Eagle Medium) medium, fetal bovine serum (FBS), phosphate buffered saline (PBS), 0.25% (*W/V*) trypsin (with 0.02% EDTA), isoprenaline (ISO) and verapamil (VRP) were purchased from Sigma, St. Louis, MO, USA. A 1% antibiotics solution (100 μ/mL penicillin and 100 μ/mL streptomycin) and collagenase type I were purchased from Life technologies, Carlsbad, USA. Fibronectin (FN) and 3-mercaptopropionic acid (1-mercaptopropionic acid, MP) were purchased from Life Technologies, USA. Mercaptopropionic acid (MPA), N-Hydroxysuccinmide (NHS) and N-(3-Dimethy lamino prop yl)-N′-ethylcarbodiimide hydrochloride (EDC, EDC) were purchased from Sigma Aldrich, USA. Concentrated sulfuric acid, 30% hydrogen peroxide and anhydrous ethanol were purchased from Sigma Aldrich. Gelatin was purchased from Sinopharm Chemical Reagent Co., Shanghai, China. Ultra-pure nitrogen gas was purchased from Changsha Xinxiang Gas Analysis Instrument Business Department, Hunan, China).

### 2.3. Main Instruments and Equipment

**Figure 1:**
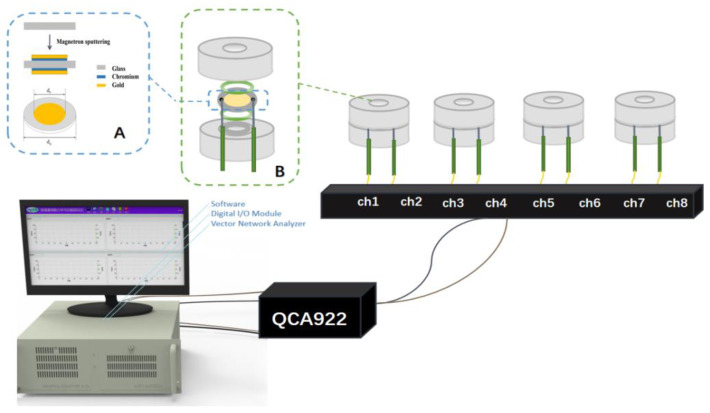
Diagram of the QCM unit ((**A**): process flow and diagram of the QCM piezoelectric resonator; (**B**): diagram of the Teflon well cell assembly).

A QCA 922 eight-channel quartz crystal microbalance (QCM) was purchased from Seiko-EG&G. The 9 MHz AT-cut crystal was custom-made with a quartz crystal diameter of 13.67 mm and an electrode diameter of 5.1 mm, with one side of the crystal in contact with the cell suspension and the other side in contact with an O-ring to form an air chamber at the bottom of the Teflon reaction cell, as shown in Figure 1A Countess TM II automatic counter (ThermoFisher), an IX71 inverted microscope (Olympus, Tokyo, Japan), a SP-300 electrochemical workstation (Bio-Logic, Rue de Vaucanson, France), a Milli-Q integrated pure/ultrapure water system and an Olympus inverted fluorescence microscope were the main instruments that were used in this study.

## 3. Methods

### 3.1. Teflon Cell Cleaning

The Teflon cell, rubber ring and screws were placed in a beaker with anhydrous ethanol and cleaned by ultrasonication for 480 s, this was repeated three times. Then, they were placed in a beaker with deionized water, and cleaned by ultrasonication for 480 s, and this was repeated two times. Then, the Teflon pool was blow dried with nitrogen gas, and placed in an ultra-clean bench and exposed to UV light for approximately 15 min.

### 3.2. Gold Electrode Pretreatment

Firstly, the surface of the gold electrode was cleaned with anhydrous ethanol and deionized water, respectively, following which, it was blow dried with nitrogen gas. The pre mixed acid solution (1:3(*v:v*)30%H_2_O_2_:H_2_SO_4_) was placed in a water batch at 80 °C, on the surface of the gold electrode (note that the mixed acid solution corrodes the conductive glue here) for 20 s, then it was rinsed with deionized water, blow dried with nitrogen, and the steps were repeated three times until the surface of the gold electrode was clean and free from contaminants. The pretreated gold electrodes were assembled into a Teflon well cell, sterile water was added and sealed with a waterproof evaporative film, moved to an ultra-clean bench and irradiated with UV light overnight.

### 3.3. Isolation and Extraction of the Primary Cardiomyocytes from Suckling Rats

Eight 2 day old SD rats were immersed in 75% alcohol until they were unconscious. Then, a small incision in the abdomen was cut, using ophthalmic scissors, the thoracic cavity was cut along the sternum. The heart was removed and placed in a pre-cooled PBS solution at 4 °C and was held in the corners of a flat dish. The residual blood was squeezed out, the atria were removed and the ventricular portion was transferred to another round dish containing PBS solution and cut into small pieces of approximately 1 mm^3^. The tissue pieces were transferred to a small beaker, the original PBS solution was aspirated and an equal volume of 2 mL each of trypsin/combination type II collagenase was added. The beaker was sealed with a sealing film and transferred to a magnetic stirrer at 37 °C for 16 r/min for 6 min, to facilitate the tissue digestion. Then, the digestion was ended by adding 2 mL of DMEM with 20% FBS, and the sample was kept aside for 10 s to allow the tissue pieces to sink to the bottom of the bottle, and the upper cell suspension was collected for subsequent use. The above steps were repeated six times until the tissue block was completely digested. The digested cell suspension was centrifuged (1000 rpm, 10 min) one to two times, resuspended in DMEM supplemented with 10% FBS and inoculated into cell culture flasks. The cell culture flask was incubated in 5% CO_2_, a 37 °C incubator for 2 h, and the unattached cell suspension was aspirated from the flask (at this time, the non-cardiomyocytes were already apposed, and the cells not yet apposed were cardiomyocytes, i.e., differential apposition method, the purity of the cardiomyocytes obtained by this method was over 90%), and the cell density was adjusted to 3 × 10^5^ cells/mL with DMEM containing 10% FBS, and 5-Bromodeoxyuridine 0.0l mmol/L (to inhibit the proliferation of non-cardiomyocytes) was added, and the culture medium was replaced after 48h.

### 3.4. Electrical Impedance Sensing (EIS) Technology Coupled with the Quartz Crystal Microbalance (QCM) Technology for Monitoring the Adhesion and Pulsation of Rat Primary Cardiomyocytes

#### 3.4.1. Screening of the Optimal Culture Conditions for the EIS Technique

1. Optimization of the cell density

Four cell densities, including 20,000, 40,000, 60,000 and 80,000 cells/per 200 μL of well-grown primary cardiomyocytes, were added to four Teflon cells equipped with bare gold electrodes, and placed in the incubator, and the impedance of the target cells was continuously monitored in the frequency range of 10 Hz to 4 MHz. The cell density suitable for the drug screening was determined by combining the cell index characteristics.

2. Optimal modification scheme

Once the optimal cell density was identified, the gold electrode was pretreated again and the Teflon cell was installed. The bare gold electrode was used as the control group, and the other three groups were modified with 0.1% collagen, 0.1% gelatin and FN, respectively.

Experimental group I: 0.1% collagen modification: 1% collagen was placed in a gold electrode cell, left overnight at 4 °C. The subsequent day, the collagen was removed and washed again with PBS, and the cell loading assay was performed.

Experimental group II: 0.1% gelatin modification: 1% gelatin was placed in the gold electrode cell, left overnight at 4 °C. The subsequent day, the gelatin was removed and washed with PBS, and the cell loading assay was performed.

Experimental group III: FN modification: 100 µL of MPA was added to 4900 µL of anhydrous ethanol and mixed well. Then, 200 µL of the solution was added to each gold electrode cell, overnight (15 h) in a dark environment. Then, 0.0069 g of NHS and 0.0575 g EDC were added to 2 mL of PBS (pH 5.5). The MPA was removed and 200 µL was added to each well for 1.5 h. The liquid was removed and sterilized with 75% alcohol for 25 min. Next, 20 µg/mL of FN was placed in PBS (pH 8.2, 20 µL 1mg/mL FN in 980 µL PBS). The alcohol was removed, the PBS was gently rinsed, and 200 µL of 20 µg/mL FN was added to each well for approximately 4 h, and the cell loading test was performed.

3. Optimal electrode thickness

Following the determination of the optimal cell density and the modification scheme, the quartz crystal chips with gold electrode thicknesses of 20 nm, 50 nm, 100 nm and 150 nm were used to evaluate the optimal electrode thickness during the adhesion of the rat primary cardiomyocytes on an electrical impedance sensing instrument.

It should be noted that the two individual optimal solutions were not necessarily optimal for each other when they were, in effect, together. In this paper, we were unable to make a more detailed experimental protocol, due to time constraints, and here we propose an optimization approach involving a sequence of 4 × 4 × 4 = 64 experimental protocols, which was performed to test each condition individually and to filter the optimal conditions.

#### 3.4.2. Monitoring the Beating of the Rat Primary Cardiomyocytes Using EIS, in Combination with the QCM

The rat primary cardiomyocytes that were growing well and beating, were added to a modified Teflon well cell in an incubator and their impedance values were continuously monitored on the EIS, at a constant frequency of 10 kHz. At the same time, the EIS and QCM were connected by wires for the continuous and simultaneous data acquisition.

The cell index (CI) [18] was introduced to evaluate the changes in the impedance, due to the cell-electrode interactions in an electrochemical impedance sensor system, in order to obtain a more uniform, intuitive and simple value. Thus, instead of the impedance values, the CI values were used to reflect the state of the cell growth, the proliferation, the cell-to-cell contact and the cell-to-substrate adhesion.

### 3.5. Data Processing and Analysis

The data collected by the EIS-based SP-300 electrochemical workstation were analyzed by Eclab software, and the raw data was replicated and fed into the Origin8.0 software and GraphPad Prism software for the data processing and analysis. The impedance values and the cell indices (CIs) were calculated using the Origin8.0 software with custom functions.

## 4. Results and Analysis

### 4.1. Optimization of the Cell Density for the Screening

**Figure 2:**
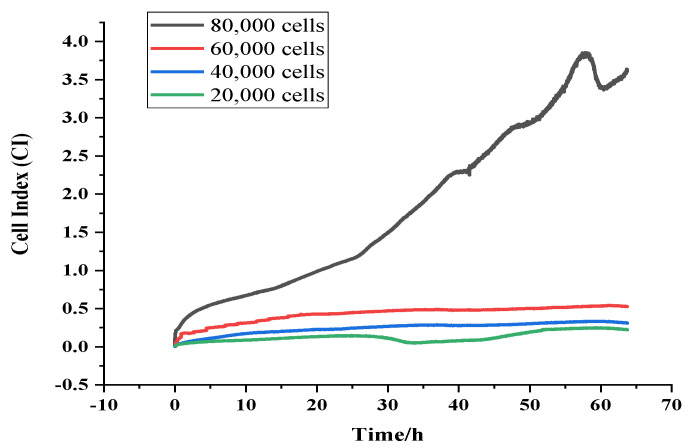
Comparison of the cell indices at different cell densities.

Figure 2 and Figure S1 (Supplementary Materials) show the comparison of the cell index (CI)-time (T) for different cell densities of rat cardiomyocytes, which was monitored in real time, by the EIS technique at frequencies ranging from 10 Hz to 4 MHz for 20,000, 40,000, 60,000 and 80,000 cells/200 μL, respectively, in the absence of interfacial modifications. As shown in Figure 2, the moment at which the rat primary cardiomyocytes began to adhere in large numbers, varied considerably between the different density conditions. The degree of adhesion was positively correlated with time, over a range of time points, and this process represented the optimal exponential growth period where the cells progressed from the suspension state to subsequent stages, such as the wall attachment, latency, proliferation and reached 70%–80% confluence. This could be due to the increased cell adhesion over time, the death of some cells, or the accumulation of metabolites in the culture medium that made the culture conditions unsuitable for cell adhesion, growth or even pulsation. As shown in Figure S1, the CI values measured at each cell density could be viewed individually. With the exception of the 80,000 cells/200 μL density, the CI values were below 1.0. for the remaining conditions, which showed a consistent trend with the CI values continuing to increase after the cells were added, indicating that the cells continued to adhere during the process. Once the adhesion was complete, some of the cells grew, reaching a peak after 50 h. Following the monitoring of several experiments, it was found that the cell adhesion ability and cell density within a certain range were positively correlated, moreover, the peak of the CI appeared between 50 h and 60 h after the addition of the cells, following which there was a significant decline. Previously, it was reported [19] that a high CI value and the response brought about by the excessive cell growth could interfere with the drug action process. In contrast, the cell index in the range of 0.5–1 indicated that the particular cell density was suitable for subsequent functional experiments. The CI value of 60,000 cells/200 μL density was around 0.5, so the cell density of 60,000 cells/(200 μL) was chosen as the optimal culture density for the rat primary cardiomyocytes, based on this principle.

### 4.2. Selection of the Optimal Modification Options

**Figure 3:**
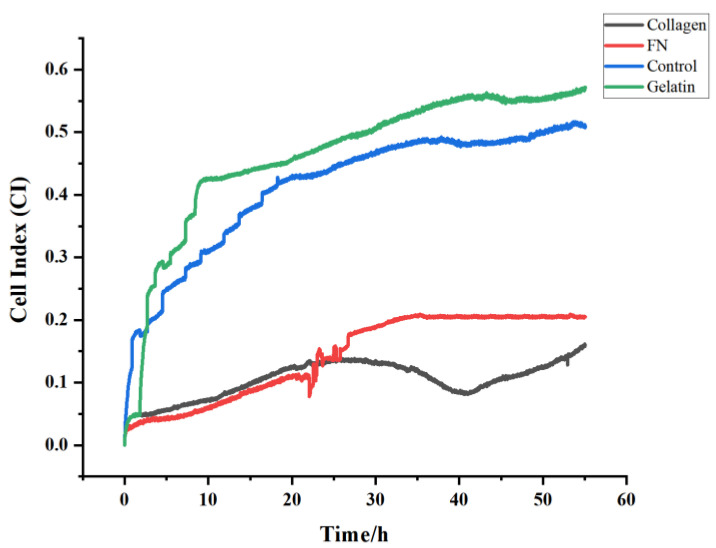
Comparison of the cell indices under different cell modification schemes.

Figure 3 and Figure S2 show the cell index (CI)-time (T) plots of the rat primary cardiomyocytes at a density of 60,000 cells, which were monitored under different cell modification schemes. As can be seen from the plots, all four conditions showed generally positive correlations in the CI-time plots, moreover, all of them showed a certain tendency of a sudden increase in the adhesion capacity at the initial stage, and some were accompanied by a certain degree of oscillation, which could be caused by voltage fluctuations or the accumulation of dead cells over the extended incubation time. According to the CI analysis, the CI values decreased from 0.53 to 0.16 after the collagen modification and to 0.23 after the FN modification, indicating that the adhesion behavior of the rat primary cardiomyocytes was inhibited by FN and the collagen modifications. However, the CI value increased, compared to the bare gold electrode with the gelatin modification, to around 0.57. This suggested that the gelatin modification scheme was more suitable than the collagen modification, the FN modification and the bare gold electrodes. Therefore, gelatin was chosen as the optimal modification method for the impedance test interface of the rat primary cardiomyocytes.

### 4.3. Optimization of the Gold Electrode Thickness

**Figure 4:**
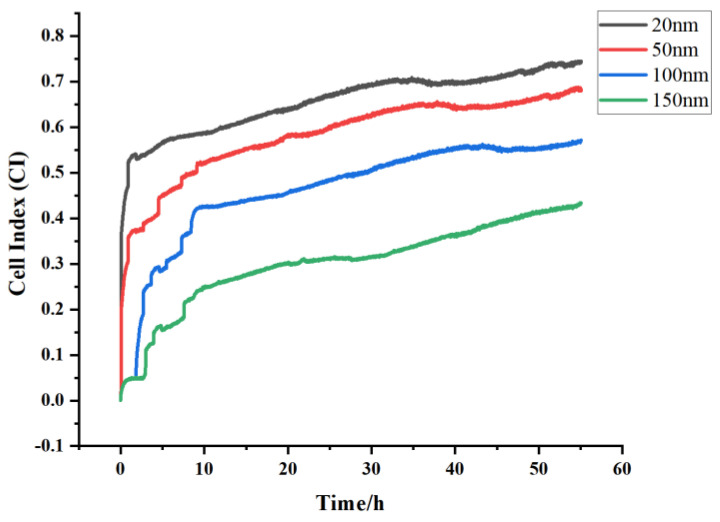
Comparison of the cell indices at different chip thicknesses.

Figure 4 and Figure S3 show the cell index-time comparisons of 60,000 rat primary cardiomyocytes monitored on the gold electrode surface of a quartz chip modified with gelatin at different gold electrode thicknesses. The results showed that the response trend of the cardiomyocytes at different thicknesses was relatively consistent, but the sensitivity of the impedance response varied with the different electrode thicknesses, and decreased with increasing electrode thickness. In the previous experiment, we used a an electrode thickness of 100 nm with a CI value of approximately 0.57, while at 20 nm and 50 nm electrode thicknesses, the CI values were 0.74 and 0.68, respectively, and the CI value dropped to around 0.43 at 150 nm electrode thickness, indicating that the electrode thickness also affects the dynamic response of the cardiomyocytes, in terms of impedance. Therefore, 20 nm was chosen as the optimum electrode thickness with a CI value between 0.5 and 1.0, which was suitable for the subsequent cell function experiments, and it had a high sensitivity.

### 4.4. Monitoring the Pulsation of the Primary Cardiomyocytes and the Drug Toxicity in Rat Cardiomyocytesm Using the Combination of EIS and QCM Methods

#### 4.4.1. Monitoring the Cardiomyocyte Pulsation

**Figure 5:**
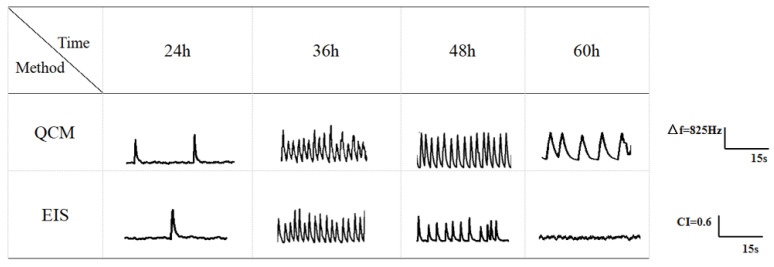
Cardiomyocyte beating signals detected by the QCM and EIS sensors at various time points. (The time duration of the signal fragment is 15 s, with a maximum frequency of 825 Hz in the QCM monitoring and a maximum CI of 0.6 in the EIS monitoring).

The beating status of the cardiomyocytes was recorded using the EIS, in combination with the QCM. Figure 5 show the beating signals detected by the QCM and EIS, at 24 h, 36 h, 48 h and 60 h, respectively, with each test lasting for 15 s. From the characteristics of the signals recorded at each time point, it could be seen that the frequency and amplitude of the beating signals of the primary neonatal rat cardiomyocytes were low at the beginning of the beating phase, and the duration of the signals was long (e.g., at 24 h). However, after a certain period of incubation, the frequency of the beating signal increased and was more regular, the duration of the signal was shorter and the amplitude of the signal gradually increased (e.g., 36 h on QCM, 48 h on EIS). Then, after a further period of incubation, the activity of the cardiomyocytes decreased, the frequency of the beating signal decreased significantly, and the duration of the signal was longer (e.g., 60 h on the QCM, 48 h on EIS), and finally, the beating signal gradually disappeared altogether (e.g., 60 h on EIS).

**Figure 6:**
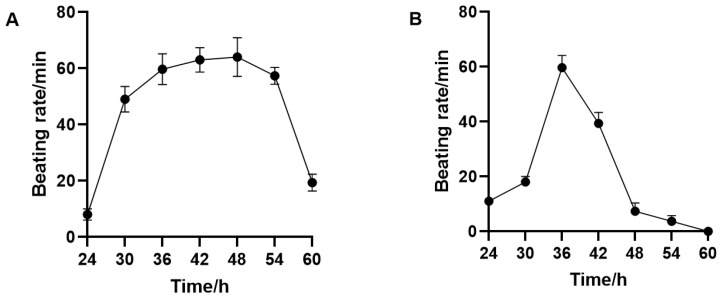
Statistical analysis of the multichannel cardiomyocyte beat frequency (n = 3) ((**A**): QCM monitoring; (**B**): ECIs monitoring).

The beating frequency of the cells was low, below 20 beats/min, during the 24 h of culture (Figure 6), and high and stable around 60 beats/min, until the 36th hour. At 60 h, due to the decrease in the cellular activity, the beating frequency also decreased gradually to around 20 beats/min. However, in the EIS impedance sensor, the cardiomyocyte beating signal showed a decrease at 40 h, and the frequency of the beating signal decreased significantly from 48 to 60 h and gradually and completely disappeared at the end. The above results were consistent with that of Wang Tianxing’s [20] study, where they monitored the impedance of the cardiomyocyte beating status in a cell-based electrical impedance sensor system, to analyze multiple relevant physiological parameters. The difference between the two methods was that the cell pulsation was maintained for a longer period of time in the QCM, reaching a stable state between 36 h and 54 h. Furthermore, the pulsation was still present at 60 h without a signal loss, probably due to the improved stability of the QCM system device, which made it more suitable for the cell growth and division, leading to a better cell state than that of the EIS system. Based on the statistical analysis, we found that the beating frequencies at each time point were similar in both methods, with similar changes and small standard deviations, indicating that primary cardiomyocytes cultured under the same condition were in a similar state between the channels and displayed a consistent behavior, which is a prerequisite for the construction of the cardiomyocyte sensors.

#### 4.4.2. Evaluation of the Drug Toxicity, Using the Combination of EIS and the QCM

To further evaluate the analytical performance of the optimized cardiomyocyte QCM sensor, two standard drugs, isoprenaline (ISO) and verapamil (VRP), were selected for the pharmacological testing. To analyze the cell index (CI) and frequency (∆f) response characteristics of the EIS and QCM, at different concentrations of ISO and VRP, and the pharmacological effects of these drugs, we extracted two characteristic parameters from the raw beat signals, namely, the beat amplitude (BA) and beat frequency (BR).

**Figure 7:**
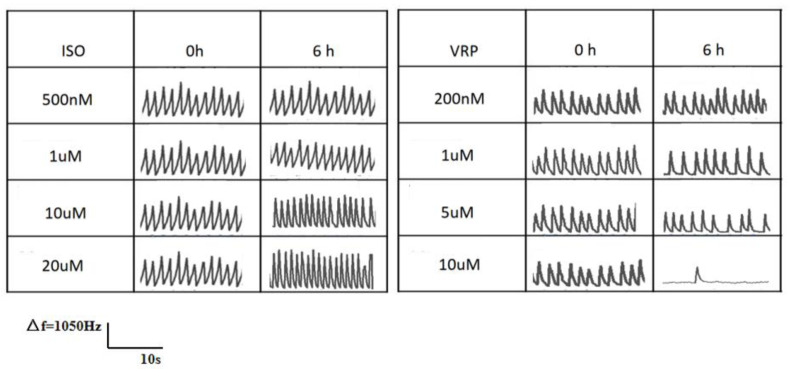
Beat signal changes in the cardiomyocyte QCM sensors after treatment with different concentrations of isoproterenol (ISO) and verapamil (VRP), with a time lapse of 10 s for the signal fragments and a maximum Δf of 1050 Hz.

**Figure 8:**
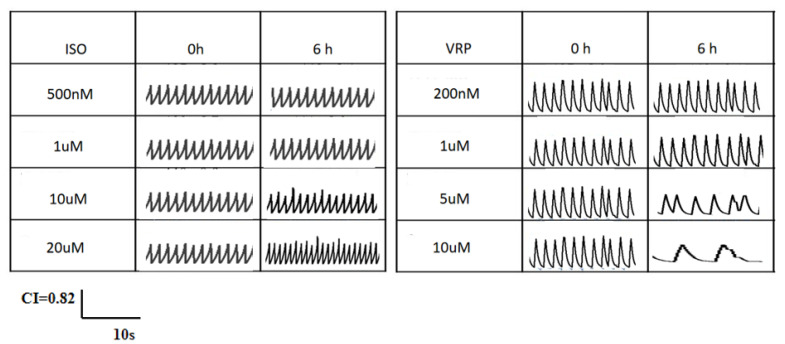
Beat signal changes in the cardiomyocyte EIS sensors after treatment with different concentrations of isoproterenol (ISO) and verapamil (VRP), with a time lapse of 10 s for the signal fragments and a maximum CI of 0.82.

ISO is a β agonist, which has been reported to accelerate the beat frequency and increase the beat amplitude of cardiomyocytes [21,22]. Verapamil is a class IV antiarrhythmic agent, an inhibitor of the calcium inward flow (slow channel blocker), which generally causes a slowing of the heart rate [23,24]. Figure 7 shows the changes in the beat-to-beat signal of a single channel of the QCM sensor in cardiomyocytes after 6 h of treatment with different concentrations of ISO and VRP. The results showed that the response of the QCM sensor to the ISO output at 500 nM and 1 µM concentrations, was relatively weak, with a slight acceleration in the beat frequency of the signal, but almost no change in the beat amplitude. The response to 10 µM and 20 µM concentrations of ISO was stronger, with a significant acceleration in the beat frequency of the signal and a small increase in the beat amplitude. In addition, the beat frequency was slightly slower and the beat amplitude was almost unchanged at 200 nM and 1µM VRP. The response to 5 µM and 10 µM VRP was also stronger, but the beat frequency of the signal was significantly slower and there was a significant decrease in the beat amplitude, especially at 10 µM VRP, where a period of pacing arrest occurred. The above results indicated that ISO enhanced the pacing function of rat cardiomyocytes and VRP weakened their pacing function, respectively, which was in accordance with their reported mechanism of action. Figure 8 shows the changes in the beating signal of the single channel of the EIS sensor in cardiomyocytes after the 6 h treatment with different concentrations of ISO and VRP, and the overall trend was consistent with the beating signal measured by the QCM.

**Figure 9:**
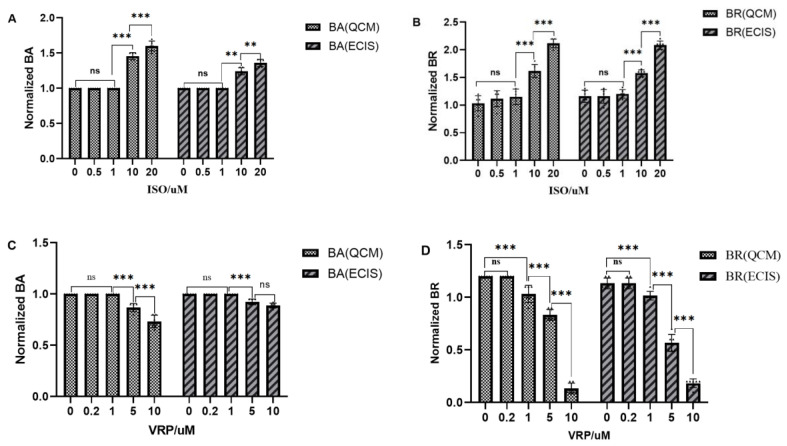
Plot showing the response characteristics of the QCM and EIS in the cardiomyocytes after 6 h of drug action (Mean ± SD, n = 6) (**A**) Response of the characteristic BA parameter to the different concentrations of ISO; (**B**) Response of the characteristic BR parameter to the different concentrations of ISO (**C**) Response of the characteristic BA parameter to the different concentrations of VRP; (**D**) Response of the characteristic BR parameter to the different concentrations of VRP. (the value bars with ns are not significant, asterisk denotes statistically significant differences ** *p* < 0.01; *** *p* < 0.001).

Further comparisons between the differences in myocardial beat signals measured by the two methods and the results of the statistical analysis of the BA and BR parameters of the QCM and EIS beat signals after 6 h of ISO and VRP, respectively, are shown in Figure 9. The results indicated that the trends measured by the two methods were similar, with neither the myocardial beat frequency nor the beat amplitude showing significant differences at lower concentrations of ISO and VRP (*p* > 0.05, n = 6), while BA and BR of the beat signals, at higher concentrations of ISO (e.g., 10 µM, 20 µM) and VRP (e.g., 1 µM, 5 µM, 10 µM), showed an increase, compared to the control and lower concentrations (*p* < 0.001). The difference between the two was that the beat signals measured on the EIS were smaller in the high concentration group, compared to those measured on the QCM, with the difference ranging between 0.1 to 0.3. The above results indicated that ISO enhanced the mechanical beating of the cardiomyocytes, while VRP decreased it and that there was a certain dose dependent effect. These results were consistent with the previously reported findings in the literature and reinforced the fact that the myocardial beating results measured by the QCM and EIS were consistent, and that the QCM method had a greater sensitivity than the EIS method, in terms of the cardiomyocyte beating tests, indicating that the QCM method may have a greater potential for the real-time monitoring of myocardial beats.

## 5. Discussion

In this study, we used a combination of EIS and QCM methods to monitor the adhesion, growth and myocardial beating function of the rat cardiomyocytes covered by the EIS in real time, by optimizing a series of conditions, including cell growth, adhesion, conditions on the EIS and dosage. In the CI-T curves, the myocardial beat frequency and beat amplitude were compared with those obtained from the QCM monitoring, to visualize the changes in the cell adhesion and the beat function, as well as before and after dosing. During the experiments, the acquisition of the beating signals from the rat primary cardiomyocytes required a certain frequency of ECIS scanning on our SP-300 electrical impedance sensing instrument. Following the repetition of the experiments and a review of the literature [16,25], we selected a fixed frequency of 10 kHz for the electrical impedance scanning of the chip covered by the rat primary cardiomyocytes. The fixed frequency was needed as it was inextricably linked to the beating cycle of the cardiomyocytes. In our extensive experimental investigations, we found that the beating effect of the cells did not reach a steady state. Thus, we also adjusted the output voltage of the ECIS in the electrical impedance sensing instrument, and found that the beating effect of the rat primary cardiomyocytes was weak when the output voltage was stabilized at 5 mV, and the current at the gold electrode was low. Moreover, the ability to capture the deformation of the trench during the beating of the cardiomyocytes was poor. We then chose to stabilize the output voltage at 20 mV, which caused an increase in the impedance values in the EIS images obtained from the CMs scanned at 10 kHz, generating a more stable myocardial pulsation curve. Additionally, in order to obtain more ideal myocardial pacing curves, we further investigated the different myocardial cell densities, the self-assembly modification conditions of the EIS chips and the different electrode thicknesses of the chips. Firstly, we know that the cell density affects the cell adhesion to a certain extent, wherein a high cell density increased the number of cells on the electrode surface for the interaction force between the cells generated, resulting in a non-uniform monolayer of cells on the electrode surface and the development of adhesion abnormalities. However, at a too little cell density, the cardiomyocytes could not achieve synchronous beating, hence the cardiac beating signal could be obtained. Finally, we selected a density of 60,000 cells/(200 μL) for the subsequent functional experiments. Secondly, the self-assembly modification allowed the cells to adhere more efficiently and tightly to the gold electrode, whereas the FN modification had a positive impact on the cells and facilitated the cell adhesion on the gold electrode carrier interface [26,27]. Collagen is a biopolymer synthesized by animal cells, whose affinity for animal cells is self-evident [28,29]. Gelatin, a typical denatured form of collagen, is a hydrolysis product of collagen and theoretically facilitates the adhesion of rat primary cardiomyocytes and enhances the sensitivity of the gold electrodes [30,31]. The results showed that the gelatin modification had a significant advantage for monitoring the myocardial beats with the EIS, potentially because the extracellular matrix of the myocardium was rich in collagen. Gelatin, an articulated form of collagen, has a more detailed spatial quaternary structure and a more flexible structural domain than collagen, which promotes the cell adhesion and proliferation, thus allowing the cardiomyocytes to adhere faster and more firmly, while FN may have a more profound effect on vegetative cells. The thickness of the EIS gold coating has been seldom explored in previous studies, conversely, this paper explores the most suitable thickness, from the perspective of studying the pulsation of cardiomyocytes. The results showed that at a thickness of 20 nm, the cell index values were more suitable for subsequent functional studies. At last, the use of the DC electrochemical impedance analysis, in conjunction with the QCM, has been reported internationally since the mid-1990s, whereas the use of electrochemical AC impedance, in conjunction with the QCM techniques, is rare and mainly used for protein adsorption, precipitation and denaturation processes, analytical applications, electrolytic processes with intermediate solubility electrode products, the monitoring of the adsorption and precipitation processes, the monitoring of the bacterial growth process and DNA oxidative damage, etc. [32]. However, the combined application of the two techniques for monitoring the cardiomyocyte pulsation, as well as drug screening, has not been reported before. In this study, we investigated the combination of the EIS and QCM methodologies, solved the problem of the signal interference between the two methods, explored the optimal conditions for the EIS method to monitor the cardiomyocyte pulsation, and obtained several parameters for the cardiomyocyte adhesion and pulsation. The experimental results showed that the results obtained by the QCM and EIS were consistent, moreover, the QCM technique had a higher sensitivity, which confirmed the reliability and validity of the QCM technique for monitoring the cardiomyocyte pulsation and drug testing.

## Data Availability

The authors confirm that the data supporting the findings of this study are available within the article.

## Supplementary Materials

The following supporting information can be downloaded at: https://www.mdpi.com/article/10.3390/bios13020198/s1, Figure S1: Comparison of the cell indices at different cell densities (Subplot). (A) Cell index plot for 20,000 cells; (B) Cell index plot for 40,000 cells; (C) Cell index plot for 60,000 cells; (D) Cell index plot for 80,000 cells; Figure S2: Comparison of the cell indices under different chip modification schemes (Subplot). (A) Cell index plot for the collagen modification; (B) Cell index plot for the FN modification; (C) Cell index plot for the bare gold electrodes; (D) Cell index plot for the gelatin modification; Figure S3: Comparison of the cell indices at different electrode thicknesses of the chips (Subplot). (A) Cell index plot at a 20 nm thickness; (B) Cell index plot at a 50 nm thickness; (C) Cell index plot at a 100 nm thickness; (D) Cell index plot at a 150 nm thickness.
