# Supplementary material for: Quartz Crystal Microbalance Technology Coupled with Impedance for the Dynamic Monitoring of the Cardiomyocyte Beating Function and Drug Screening
Source: Biosensors (Basel). 2023 Jan 28;13(2):198. doi: 10.3390/bios13020198 (PMC9953959; doi:10.3390/bios13020198)
Supplement: Supplementary file 1 [file biosensors-13-00198-s001.zip › biosensors-2082349-supplementary.pdf]

Supplementary Figures

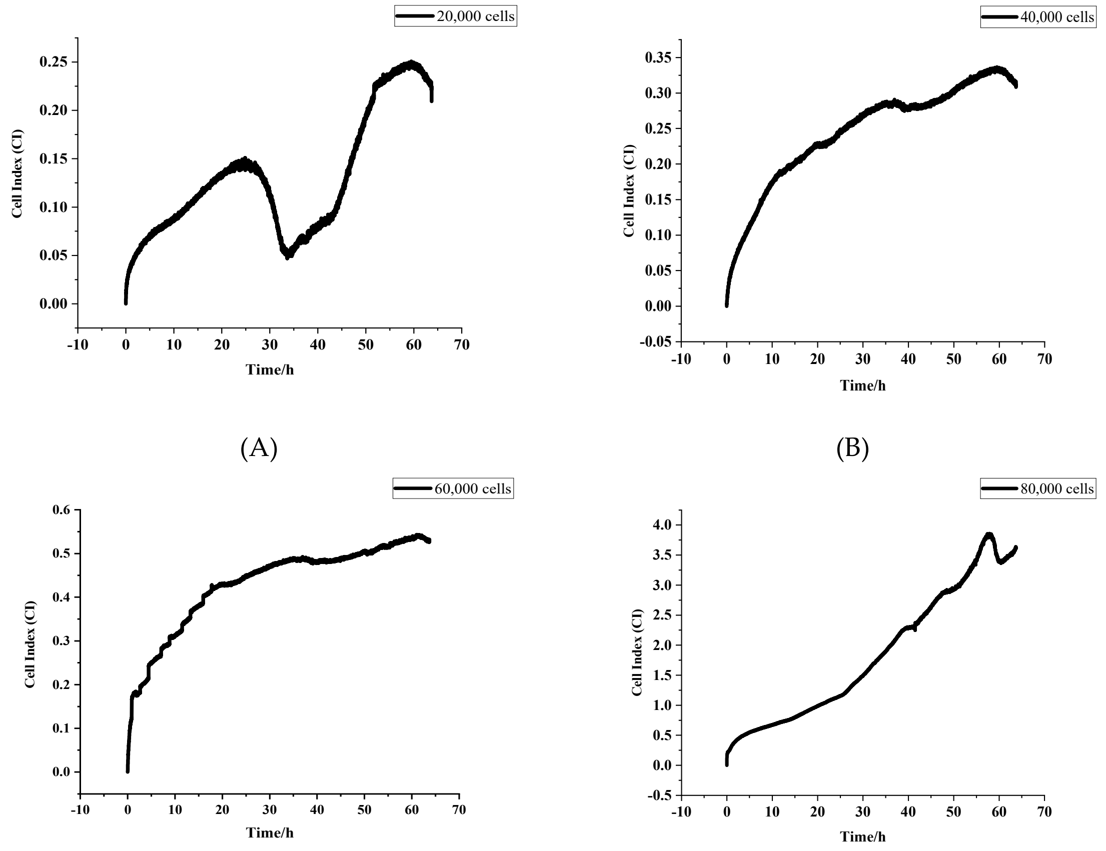

(A) (B)

(C) (D)

Figure S1. Comparison of cell indices at different cell densities (Subplot). (A)Cell index plot for 20,000 cells; (B) Cell index plot for 40,000 cells; (C)Cell index plot for 60,000 cells; (D) Cell index plot for 80,000 cells.

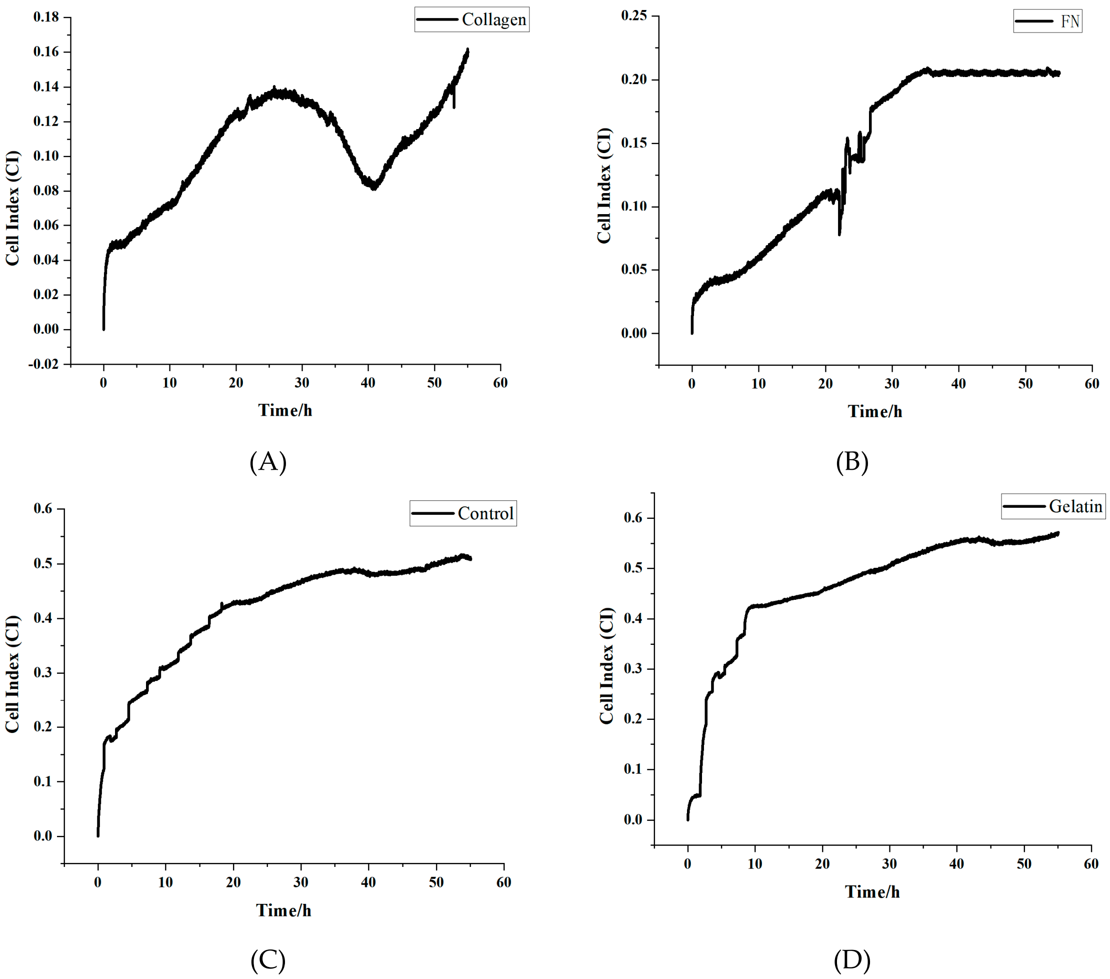

Figure S2. Comparison of cell indices under different chip modification schemes (Subplot). (A)Cell index plot for collagen modification; (B) Cell index plot for FN modification; (C) Cell index plot for bare gold electrodes; (D)Cell index plot for gelatin modification.

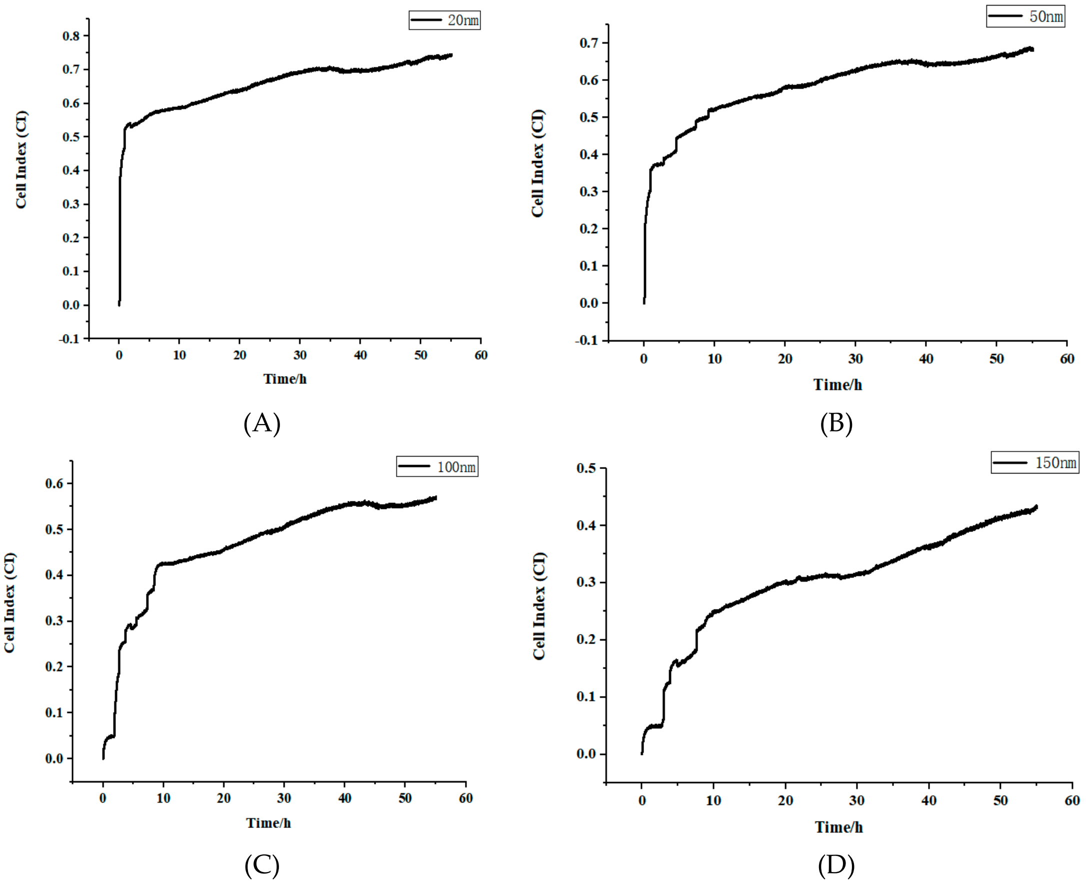

Figure S3. Comparison of cell indices at different chip thicknesses (Subplot). (A)Cell index plot at 20nm thickness; (B) Cell index plot at 50nm thickness; (C) Cell index plot at 100nm thickness; (D) Cell index plot at 150nm thickness.
